# Decoupling facial motion features and identity features for micro-expression recognition

**DOI:** 10.7717/peerj-cs.1140

**Published:** 2022-11-14

**Authors:** Tingxuan Xie, Guoquan Sun, Hao Sun, Qiang Lin, Xianye Ben

**Affiliations:** School of Information Science and Engineering, Shandong University, Qingdao, Shandong, China

**Keywords:** Micro-expression recognition, Deep learning, Feature decoupling, Facial motion features, Identity features

## Abstract

**Background:**

Micro-expression is a kind of expression produced by people spontaneously and unconsciously when receiving stimulus. It has the characteristics of low intensity and short duration. Moreover, it cannot be controlled and disguised. Thus, micro-expression can objectively reflect people’s real emotional states. Therefore, automatic recognition of micro-expressions can help machines better understand the users’ emotion, which can promote human-computer interaction. What’s more, micro-expression recognition has a wide range of applications in fields like security systems and psychological treatment. Nowadays, thanks to the development of artificial intelligence, most micro-expression recognition algorithms are based on deep learning. The features extracted by deep learning model from the micro-expression video sequences mainly contain facial motion feature information and identity feature information. However, in micro-expression recognition tasks, the motions of facial muscles are subtle. As a result, the recognition can be easily interfered by identity feature information.

**Methods:**

To solve the above problem, a micro-expression recognition algorithm which decouples facial motion features and identity features is proposed in this paper. A Micro-Expression Motion Information Features Extraction Network (MENet) and an Identity Information Features Extraction Network (IDNet) are designed. By adding a Diverse Attention Operation (DAO) module and constructing divergence loss function in MENet, facial motion features can be effectively extracted. Global attention operations are used in IDNet to extract identity features. A Mutual Information Neural Estimator (MINE) is utilized to decouple facial motion features and identity features, which can help the model obtain more discriminative micro-expression features.

**Results:**

Experiments on the SDU, MMEW, SAMM and CASME II datasets were conducted, which achieved competitive results and proved the superiority of the proposed algorithm.

## Introduction

Micro-expressions are brief and involuntary facial expressions which are generated by human facial motions. Compared to obvious macro-expressions, micro-expressions have short duration and low intensity (Ekman & Friesen, 1969) as facial motions are subtle and emerge in only a few facial regions. As micro-expression (ME) is involuntary, it has a wide range of applications in various domains such as public safety and psychological treatment. Therefore, the research of micro-expression recognition (MER) can help machines better understand human emotion and promote human-computer interaction. Several ME datasets were collected in recent years, such as CASME II (Yan et al., 2014) and SAMM (Davison et al., 2018). A new ME dataset, MMEW (Ben et al., 2022a), was released to public by our team, which contains both macro- and micro-expressions sampled from the same subjects. Although these datasets were selected under strictly controlled environment, MEs are still extremely difficult to recognize. The process of MER can be divided into two stages: feature extraction and classification. Many different automatic MER methods (Zhao & Pietikäinen, 2007; Wang et al., 2014; Lu et al., 2014; Liu et al., 2015; Liu, Li & Lai, 2021; Happy & Routray, 2017; Liong et al., 2014; Khor et al., 2018; Van Quang, Chun & Tokuyama, 2019; Xia et al., 2019; Verma et al., 2020; Li, Huang & Zhao, 2020; Xia et al., 2020; Chen et al., 2022; Li et al., 2022; Xu, Zhang & Wang, 2017; Lei et al., 2020; Xie et al., 2020; Peng et al., 2018; Ben et al., 2022b; Mao et al., 2022; Wei et al., 2022) have been proposed in the past years. We can categorize them roughly into two groups: the traditional algorithms and the deep learning-based algorithms.

As for traditional algorithms, the Local Binary Pattern from Three Orthogonal Planes (LBP-TOP) (Zhao & Pietikäinen, 2007) which describes dynamic textures was first applied to ME. To solve the sensitivity problem of global changes in LBP-TOP, Local Binary Patterns with Six Intersection Points (LBP-SIP) (Wang et al., 2014) was proposed. Lu et al. (2014) proposed a Delaunay-based temporal coding model (DTCM), which can encode texture variations of facial muscle motions. In addition, the main directional mean optical-flow (MDMO) proposed by Liu et al. (2015) is also a representative spatiotemporal descriptor, which is a kind of optical-flow method. Liu, Li & Lai (2021) then further added MDMO into the classic graph regularized sparse coding to generate a sparse MDMO feature. Besides MDMO, Xu, Zhang & Wang (2017) proposed a facial dynamics map (FDM) feature, which uses an iterative optimal strategy to calculate the principal optical flow direction of spatiotemporal cuboid obtained from divided ME sequences to better represent the local facial dynamics. In addition, Happy & Routray (2017) proposed a fuzzy histogram of optical flow orientations (FHOFO) for MER, which constructs angular histograms from optical flow vector orientations using histogram fuzzification to encode the temporal pattern of MEs. Liong et al. (2014) proposed a novel optical strain weighted feature extraction scheme for MER, which extracts weighted spatiotemporal information from blocked optical-flow maps. Such hand-crafted features are then sent into classifiers such as support vector machine or k-nearest neighbor.

In addition to above mentioned traditional algorithms, deep learning methods that can automatically extract deep features have also been applied recently to MER tasks and have achieved competitive performances. Khor et al. (2018) proposed an enriched long-term recurrent convolutional network (ELRCN), which uses two kinds of feature sequences, namely optical-flow and optical strain, to extract ME features. Van Quang, Chun & Tokuyama (2019) presented a network for MER which adopts CapsuleNet architecture as the main component and takes only the apex frame as input. Xia et al. (2019) used spatiotemporal recurrent convolutional networks along with balanced loss and some data augmentation strategies to extract micro-expression features. Inspired by ConvNet (Tompson et al., 2017) and ResNet (He et al., 2016), Verma et al. (2020) proposed a Lateral Accretive Hybrid Network (LEARNet), which captures facial motion information in every single ME frame to generate a dynamic representation of MEs. Li, Huang & Zhao (2020) proposed a novel MER model using only apex frame by learning joint local and global information, wherein the apex frame is located by estimating pixel-level change rates in the frequency domain. Xia et al. (2020) discovered that shallower-architecture and lower-resolution input data can help the model achieve better performance in cross-database MER tasks and correspondingly proposed a recurrent convolutional network (RCN). Chen et al. (2022) proposed a block division convolutional network (BDCNN), which extracts deep features from four kinds of optical-flow features generated from onset frame and apex frame, wherein the optical-flow maps are divided into several small blocks in order to more effectively locate the ME movements. Li et al. (2022) proposed an unsupervised cross-database micro-expression recognition method based on distribution adaptation, which consists of source domain selection model and adaptive distribution alignment model. Lei et al. (2020) used video motion amplification technology based on transfer learning to amplify ME motions, and proposed a graph temporal convolutional network (Graph-TCN) to extract deep features. Xie et al. (2020) proposed an AU-assisted graph attention convolutional network (AU-GACN), which takes the corresponding relationships between MEs and action units into consideration, and designed an AU Intensity Controllable Generative Adversarial Nets (AU-ICGAN) to overcome the problem of limited and unbalanced datasets. Peng et al. (2018) first proposed to use transfer learning in MER, which pre-trains deep model on macro-expression datasets with large size and then on micro-expression datasets. Ben et al. (2022b) first proposed a MER method using dataset alignment and active learning model and proved that active transfer learning can be successfully applied on MER tasks. Mao et al. (2022) explored the MER under real-world occlusion conditions and proposed a Region-inspired Relation Reasoning Network (RRRN) to capture the complementary relationship of facial areas. Wei et al. (2022) designed a novel Attention-based Magnification-Adaptive Network (AMAN) that can be adaptive to different magnification levels for MER.

As the current collection environment and construction method of micro-expression datasets are strictly controlled, the interferences of illumination, occlusion and view can be ignored. Due to the strong feature extraction ability of deep learning models, the extracted features inevitably contain both facial motion features and other interfering features. Thus, identity feature information becomes the biggest interference in MER tasks. To solve this problem, we propose a novel MER algorithm which decouples facial motion features and identity features. A dual branch neural network is specially designed, which consists of two branches, namely MENet and IDNet. MENet is used to extract ME features while IDNet is responsible for identity feature extraction. A Mutual Information Neural Estimator (MINE) is then utilized to decouple facial motion features and identity features, which can help the model obtain more discriminative ME features. Four successive training stages are designed to guarantee the effectiveness of the extracted features.

## Materials and Methods

### Methodology

**Figure 1:**
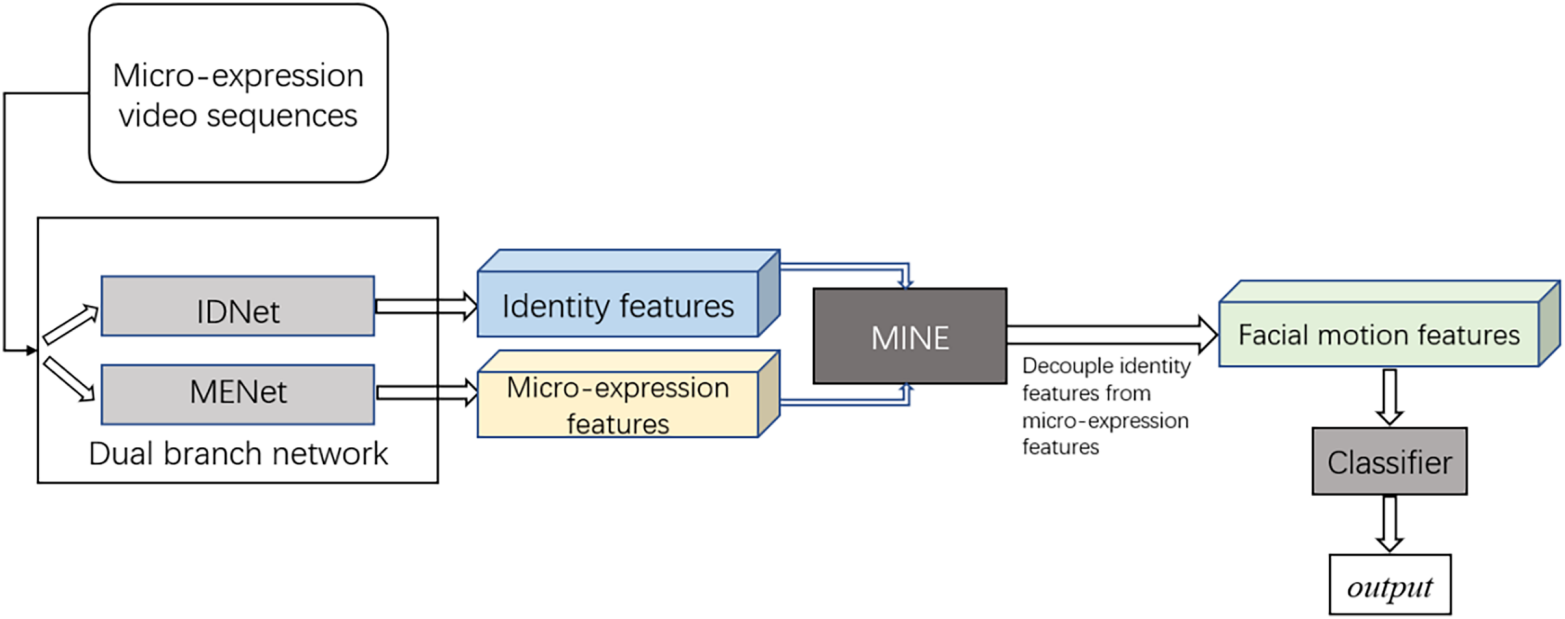
The proposed methodology and experimental design.

The proposed methodology and experimental design are shown in Fig. 1. At present, most MER tasks are based on deep learning technology. The features extracted by deep learning model from the micro-expression video sequences mainly contain facial motion information and identity feature information due to the strong extraction ability of deep model. However, in micro-expression recognition tasks, the motions of facial muscles are subtle due to the characteristics of micro-expressions while the identity information is more obvious. Moreover, the identity feature information has no contribution to the final recognition result. Thus, identity feature can be viewed as a kind of interference to MER tasks. To solve this problem, identity features are decoupled from the features learnt by deep model in the proposed method to alleviate the negative impact. Specifically, the identity features learnt by IDNet are decoupled from the features learnt by MENet using MINE module in our experiments.

### Dual branch neural network

**Figure 2:**
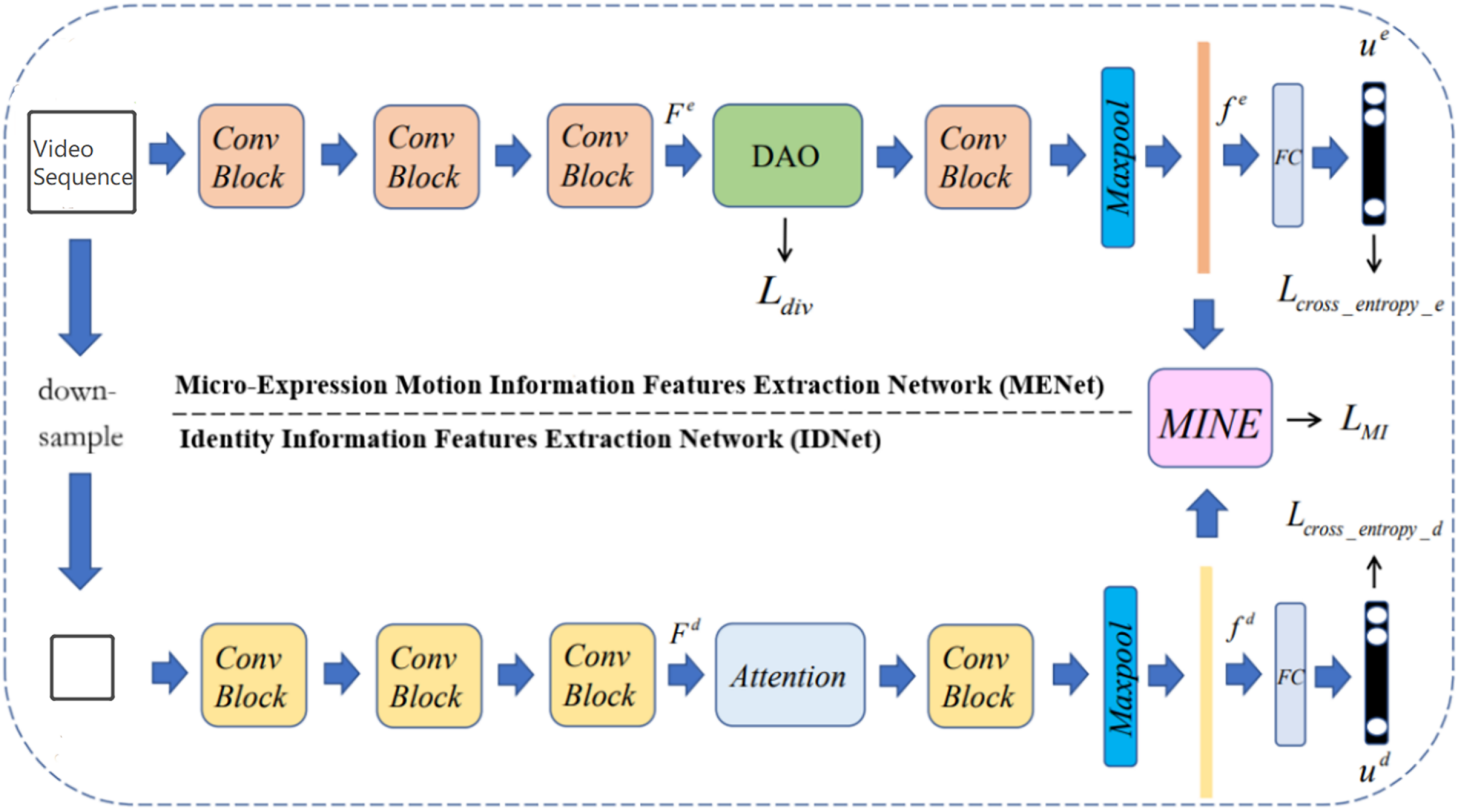
Framework of the proposed dual branch neural network.

The framework of the proposed dual branch neural network is shown in Fig. 2, which contains two neural network branches, namely Micro-Expression Motion Information Features Extraction Network (MENet) and Identity Information Features Extraction Network (IDNet).

### Network architecture

Considering the good performances of 3DCNN in different computer vision tasks, the feature extraction modules in MENet and IDNet are two modified versions of 3D-ResNet18. Their architectures are shown in Tables 1 and 2, respectively.

**Table 1:** The architecture of the modified 3D-ResNet18 in MENet.

Name	Operation	Kernel	Stride	Padding	Output channel
conv0	3DConv	{7 × 7 × 7} × 1	{1 × 2 × 2} × 1	{3 × 3 × 3} × 1	64
	Maxpool	{3 × 3 × 3} × 1	{1 × 2 × 2} × 1	{1 × 1 × 1} × 1	64
Conv_1	3DConv	$\left\{ \matrix{3 \times 3 \times 3 \hfill \cr 3 \times 3 \times 3 \hfill} \right\} \times 2$	$\left\{ \matrix{1 \times 1 \times 1 \hfill \cr 1 \times 1 \times 1 \hfill} \right\} \times 2$	$\left\{ \matrix{1 \times 1 \times 1 \hfill \cr 1 \times 1 \times 1 \hfill} \right\} \times 2$	64
Conv_2	3DConv	$\left\{ \matrix{3 \times 3 \times 3 \hfill \cr 3 \times 3 \times 3 \hfill} \right\} \times 2$	$\left\{ \matrix{1 \times 2 \times 2 \hfill \cr 1 \times 1 \times 1 \hfill} \right\} \times 1$ $\left\{ \matrix{1 \times 1 \times 1 \hfill \cr 1 \times 1 \times 1 \hfill} \right\} \times 1$	$\left\{ \matrix{1 \times 1 \times 1 \hfill \cr 1 \times 1 \times 1 \hfill} \right\} \times 2$	128
Conv_3	3DConv	$\left\{ \matrix{3 \times 3 \times 3 \hfill \cr 3 \times 3 \times 3 \hfill} \right\} \times 2$	$\left\{ \matrix{1 \times 2 \times 2 \hfill \cr 1 \times 1 \times 1 \hfill} \right\} \times 1$ $\left\{ \matrix{1 \times 1 \times 1 \hfill \cr 1 \times 1 \times 1 \hfill} \right\} \times 1$	$\left\{ \matrix{1 \times 1 \times 1 \hfill \cr 1 \times 1 \times 1 \hfill} \right\} \times 2$	256
Conv_4	3DConv	$\left\{ \matrix{3 \times 3 \times 3 \hfill \cr 3 \times 3 \times 3 \hfill} \right\} \times 2$	$\left\{ \matrix{1 \times 1 \times 1 \hfill \cr 1 \times 1 \times 1 \hfill} \right\} \times 2$	$\left\{ \matrix{1 \times 1 \times 1 \hfill \cr 1 \times 1 \times 1 \hfill} \right\} \times 2$	512
Maxpool, FC, *softmax*					

**Table 2:** The architecture of the modified 3D-ResNet18 in IDNet.

Name	Operation	Kernel	Stride	Padding	Output channel
conv0	3DConv	{1 × 7 × 7} × 1	{1 × 2 × 2} × 1	{0 × 3 × 3} × 1	64
	Maxpool	{1 × 3 × 3} × 1	{1 × 2 × 2} × 1	{0 × 1 × 1} × 1	64
Conv_1	3DConv	$\left\{ \matrix{1 \times 3 \times 3 \hfill \cr 1 \times 3 \times 3 \hfill} \right\} \times 2$	$\left\{ \matrix{1 \times 1 \times 1 \hfill \cr 1 \times 1 \times 1 \hfill} \right\} \times 2$	$\left\{ \matrix{0 \times 1 \times 1 \hfill \cr 0 \times 1 \times 1 \hfill} \right\} \times 2$	64
Conv_2	3DConv	$\left\{ \matrix{1 \times 3 \times 3 \hfill \cr 1 \times 3 \times 3 \hfill} \right\} \times 2$	$\left\{ \matrix{1 \times 2 \times 2 \hfill \cr 1 \times 1 \times 1 \hfill} \right\} \times 1$ $\left\{ \matrix{1 \times 1 \times 1 \hfill \cr 1 \times 1 \times 1 \hfill} \right\} \times 1$	$\left\{ \matrix{0 \times 1 \times 1 \hfill \cr 0 \times 1 \times 1 \hfill} \right\} \times 2$	128
Conv_3	3DConv	$\left\{ \matrix{1 \times 3 \times 3 \hfill \cr 1 \times 3 \times 3 \hfill} \right\} \times 2$	$\left\{ \matrix{1 \times 2 \times 2 \hfill \cr 1 \times 1 \times 1 \hfill} \right\} \times 1$ $\left\{ \matrix{1 \times 1 \times 1 \hfill \cr 1 \times 1 \times 1 \hfill} \right\} \times 1$	$\left\{ \matrix{0 \times 1 \times 1 \hfill \cr 0 \times 1 \times 1 \hfill} \right\} \times 2$	256
Conv_4	3DConv	$\left\{ \matrix{1 \times 3 \times 3 \hfill \cr 1 \times 3 \times 3 \hfill} \right\} \times 2$	$\left\{ \matrix{1 \times 1 \times 1 \hfill \cr 1 \times 1 \times 1 \hfill} \right\} \times 2$	$\left\{ \matrix{0 \times 1 \times 1 \hfill \cr 0 \times 1 \times 1 \hfill} \right\} \times 2$	512
Maxpool, FC, *softmax*					

The vanilla 3D-ResNet18 network consists of several similar blocks, including basic block and bottleneck block. To tackle with the problems of overfitting and the lack of datasets in micro-expression recognition tasks, both MENet and IDNet use the modified basic block to extract corresponding features. The MENet is a deep neural network containing several 3DCNN layers, namely 3D convolutional neural networks, which can be trained to capture spatio-temporal features. As facial motion feature is a kind of spatio-temporal feature containing important and discriminative information of micro-expressions, 3DCNN can be utilized to extract these features. In particular, a DAO module was added into the MENet, which can help MENet extract complementary information from different facial regions. The DAO module is discussed in detail in the next section: Attention Modules. For IDNet, as the extraction of identity feature information mainly focuses on spatial domain, the first parameter of all convolutional kernels in IDNet is set to 1. Parameters of stride and padding are also modified to keep the number of micro-expression image sequences fixed, so that the identity features can be better extracted. Compared to the vanilla 3D-ResNet18 network, the convolutional kernels in MENet are unchanged to effectively extract spatio-temporal features, while the parameters of stride and padding in MENet are modified as the same as IDNet to keep the number of micro-expression image sequences fixed, which is also a preparation for feeding the features into MINE. The output features of the 3rd modified basic block of each branch are sent into their respective attention module, after which is the final, namely the 4th basic block, whose output features are then fed into the MINE to calculate the mutual information loss.

### Attention modules

**Figure 3:**
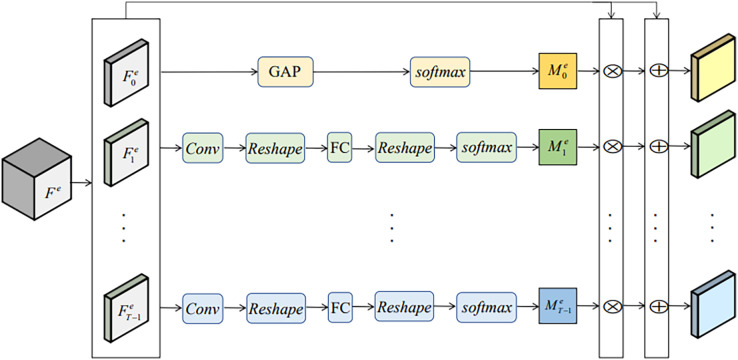
DAO module.

The facial motions of micro-expressions mainly appear in some specific regions such as eyebrows and eyelids. Although the modified 3D-ResNet18 network can effectively extract spatio-temporal features, the micro-expression features could be concentrated in some representative ROIs. To better obtain the micro-expression features, the diverse attention operation (DAO) (Hou et al., 2021) module is added in MENet to excavate more such ROIs and extract complementary information from different facial regions. DAO contains multiple attention branches, as is shown in Fig. 3. Its input is ${{F}^{{e}}}$, ${{F}^{{e}}} \in {{R}^{{C} \times {T} \times {H} \times {W}}}$, where C denotes the number of channels and T denotes the number of features. In computer vision classification tasks, the intensity of each pixel in the self-attention map is proportional to the discriminant power of the feature (Choe & Shim, 2019). Thus, DAO uses global average pooling to generate a self-attention feature map:

(1) $$\matrix{ {M_0^e = softmax\left( {\displaystyle{1 \over C}\mathop {\sum} _{c = 1}^C {{\left( {F_0^e} \right)}_c}} \right) } \cr }$$

where $M_0^e$ is the generated self-attention feature map, $softmax$ means softmax operation, $C$ denotes the total number of channels and $F_0^e$ denotes the first feature map in direction T of ${{F}^{{e}}}$.

**Figure 4:**
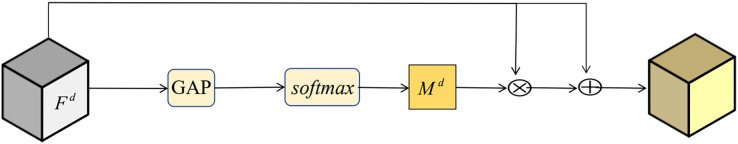
Attention module in IDNet.

Multiple parallel attention branches are then introduced to excavate different facial regions, which generate their respective attention feature maps $M_k^e \in {{R}^{H \times W}},{\rm \; }k \in \left\{ {1, \ldots ,T - 1} \right\}$. To guide DAO module to activate different facial regions and generate discriminative attention maps, divergence regularization term is introduced to calculate the difference between two attention maps:

(2) $$\matrix{ {DIV\left( {M_k^e\left| {M_l^e} \right.} \right) = 1 - sim\left( {M_k^e,M_l^e} \right) } \cr }$$

where sim (A, B) calculates the similarity between A and B. Any distance measurement that describes similarity can be applied here. In this paper we use dot-product similarity (Wang et al., 2018) due to its simplicity. Generation of self-attention maps is very helpful for IDNet to extract more robust identity features. As shown in Fig. 4, the self-attention module in IDNet is equivalent to the self-attention branch in DAO.

### Mutual information neural estimator

Features extracted by MENet inevitably contain both facial motion features and other interfering features. Considering the current collection environment and construction method of micro-expression datasets, the interferences of illumination, occlusion and view can be ignored. Thus, identity feature information becomes the biggest interference in micro-expression recognition tasks. To better obtain facial motion features, the correlation between micro-expression motion features ${{f}^{{e}}}$ and identity features ${{f}^{{d}}}$ should be minimized. Kullback-Leibler divergence can be used to decrease the correlation between two probability distributions.

Inspired by Belghazi et al. (2018) and Ruan et al. (2022), we use mutual information to measure the correlation between ${f^e}$ and ${f^d}$. Specifically, in this paper we use Mutual Information Neural Estimator (MINE), which is a neural network based on KL divergence and Donsker–Varadhan representation. The mutual information estimated by MINE can be formulated as:

(3) $$I\left( {{F^d};{F^e}} \right) = {{\rm {\cal D}}_{KL}}\left( {{P_{{F^d}{F^e}}} \Vert {P_{{F^d}}} \otimes {P_{{F^e}}}} \right)\matrix{ { \ge {E_{{P_{{F^d}}} \otimes {P_{{F^e}}}}}\left[ {{T_\theta }\left( {{f^d},{f^e}} \right)} \right] - \log \left( {{E_{{P_{{F^d}}} \otimes {P_{{F^e}}}}}\left[ {{e^{{T_\theta }\left( {{f^d},{f^e}} \right)}}} \right]} \right)} \cr }$$

where $\otimes$ denotes product function, ${P_{{F^d}{F^e}}}$ is the joint distribution of ${F^d}$ and ${F^e}$, ${T_\theta }$ is neural network MINE with parameters $\theta$. The structure of MINE is shown in Table 3.

**Table 3:** Mutual information neural estimator.

Name	Operation	Output dimension
Concatenation	Concatenate ${f^e}$ and ${f^d}$	512
FC (64)	Full connection	64
*Leaky ReLU*	Nonlinear activation	64
FC (1)	Full connection	1
*Leaky ReLU*	Nonlinear activation	1

### Optimization method

To optimize the dual branch neural network proposed in “Methodology”, multiple loss functions and four training stages are designed. Different loss functions are used in two branches and the overall training is separated into four stages to guarantee the effectiveness of both network branches.

### Loss functions

Four different loss functions are designed to optimize the network.

#### Cross-entropy loss in IDNet

The cross-entropy loss function used to measure the difference between the predicted identity $u_i^d$ and the true identity $y_i^d$ in IDNet can be written as:

(4) $$\matrix{ {{L_{cross\_entropy\_d}} = - \mathop {\sum} _{i = 1}^{{N^d}} y_i^d\log \left( {softmax\left( {u_i^d} \right)} \right)} \cr }$$

where $i \in \left\{ {1,2,...,{N^d}} \right\}$, ${N^d}$ is the total number of identities.

#### Cross-entropy loss in MENet

The cross-entropy loss function used to measure the difference between the predicted micro-expression class $u_j^e$ and the true micro-expression class $y_j^e$ in MENet can be written as:

(5) $$\matrix{ {{L_{cross\_entropy\_e}} = - \mathop {\sum}_{j = 1}^{{N^e}} y_j^e\log \left( {softmax\left( {u_j^e} \right)} \right) } \cr }$$

where $j \in \left\{ {1,2,...,{N^e}} \right\}$, ${N^e}$ is the total number of classes of micro-expressions.

#### Divergence loss function

To improve the ability of DAO module to extract discriminative features, divergence loss function is used, which can be written as:

(6) $$\matrix{ {{L_{div}} = \displaystyle{{ - 1} \over {T - 1}}\mathop {\sum}_{k = 2}^T \left( {\displaystyle{1 \over {k - 1}}\mathop {\sum}_{l = 1}^{k - 1} DIV\left( {M_k^e\left| {M_l^e} \right.} \right)} \right) } \cr }$$

where the formulation of $DIV\left( {M_k^e\left| {M_l^e} \right.} \right)$ is shown as Eq. (2).

When two attention branches in DAO concentrate on the same or similar region, the generated attention maps will have a low diversity value, which leads to a high divergence loss. In this way, more discriminative features from different facial regions can be captured by MENet.

#### Mutual information loss function

After the pre-training of IDNet, identity features ${f^d}$ can be extracted. To maximize the difference between ${f^d}$ and ${f^e}$, *i.e.*, to better decouple identity features from micro-expression features, mutual information loss function is utilized, which can be written as:

(7) $$\matrix{ {{L_{MI}} = I\left( {{F^d};{F^e}} \right) \approx {T_\theta }\left( {{f^d},{f^e}} \right) - \log \left( {{e^{{T_\theta }\left( {{f^d},{f^e}} \right)}}} \right)} \cr }$$

### Training strategy

As is shown in Table 4, four training stages are designed to guarantee the effectiveness of the extracted identity features, which helps the MENet to avoid the interference from identity information. The four separated training stages are conducted successively, from 1 to 4. The joint loss function in stage 2 and 4 can be formulated as:

**Table 4:** Training process.

Training stage	Target	Loss functions
1	IDNet	${L_{cross\_entropy\_d}}$
2	MENet	${L_{cross\_entropy\_e }}$, ${L_{div}}$, ${L_{MI}}$
3	IDNet	${L_{cross\_entropy\_d}}$, ${L_{MI}}$
4	MENet	${L_{cross\_entropy\_e}}$, ${L_{div}}$, ${L_{MI}}$

(8) $$\matrix{ {{L_{total}} = {L_{cross\_entropy\_e}} + {\lambda _{1,1}}{L_{div}} + {\lambda _{1,2}}{L_{MI}}}}$$

The joint loss function in stage 3 can be formulated as:

(9) $$\matrix{ {{L_{stage3}} = {L_{cross\_entropy\_d}} + {\lambda _2}{L_{MI}}} \cr }$$

### Datasets

The SDU dataset is collected, labelled and constructed by our research team in the laboratory of Jinan campus of Shandong University. The SDU dataset has not been released to public yet. The 73 subjects are teachers and students of the school and 855 micro-expression samples are collected. The mean age of subjects is 22.0 years old. The videos of SDU dataset have a frame rate of 50 fps. Image sequences have a resolution of 1,024 × 1,024 and the resolution of facial region is 600 × 600. There are six classes of micro-expressions labelled by FACS and emotion tags: happiness, surprise, disgust, sadness, fear and anger.

The MMEW (Ben et al., 2022a) dataset is also collected, labelled and constructed by our research team, which was published in 2021. The MMEW dataset includes two parts: MMEW macro-expression dataset and MMEW micro-expression dataset. The experiments in this paper only use MMEW micro-expression dataset. The 36 subjects are teachers and students of the school and 300 micro-expression samples are collected. The mean age of subjects is 22.4 years old. The videos of MMEW dataset have a frame rate of 90 fps. Image sequences have a resolution of 1,920 × 1,080 and the resolution of facial region is 400 × 400. There are seven classes of micro-expressions: happiness, surprise, disgust, sadness, fear, anger and others.

The SAMM (Davison et al., 2018) dataset was recorded at 200 fps from a group of 32 subjects with a mean age of 33.24. It consisted of seven basic emotions: happiness, surprise, disgust, sadness, fear, anger and others. The CASME II (Yan et al., 2014) dataset was recorded at 200 fps and contained five emotion classes: happiness, disgust, repression, surprise, and others, from 26 participants with a mean age of 22.03.

### Experimental settings

In our experiments, five-class classification experiments are conducted on SAMM dataset and CASME II dataset, six-class classification experiments are carried out on SDU dataset and MMEW dataset. Five-fold cross-validation method is used for all datasets. The experiments are all carried out using the proposed dual branch deep neural network and the four training stages are performed one by one. All experimental settings and training strategy are discussed as above. The operation system used in the experiments were Ubuntu 16.04, the GPU used is GTX 1080 with CUDA 11.0. The parameters of the neural network are discussed in the next subsection.

Different modules and loss functions are removed from the designed network to conduct ablation experiments. The ablation study is conducted on SDU dataset and the experimental settings are the same as above.

### Parameter settings

In our experiments, the input images for MENet have a resolution of 256 × 256, while the IDNet takes in downsampled images with resolution of 128 × 128. Adam is used as the optimizer for both MENet and IDNet, and the learning rate is set to 0.0001. The batch size for training model is set to 32. Maximum number of iterations of MENet and IDNet is set to 300 and 100, respectively. ${\lambda _{1,1}} = {\lambda _{1,2}} = {\lambda _2} = 0.1$.

## Results

**Figure 5:**
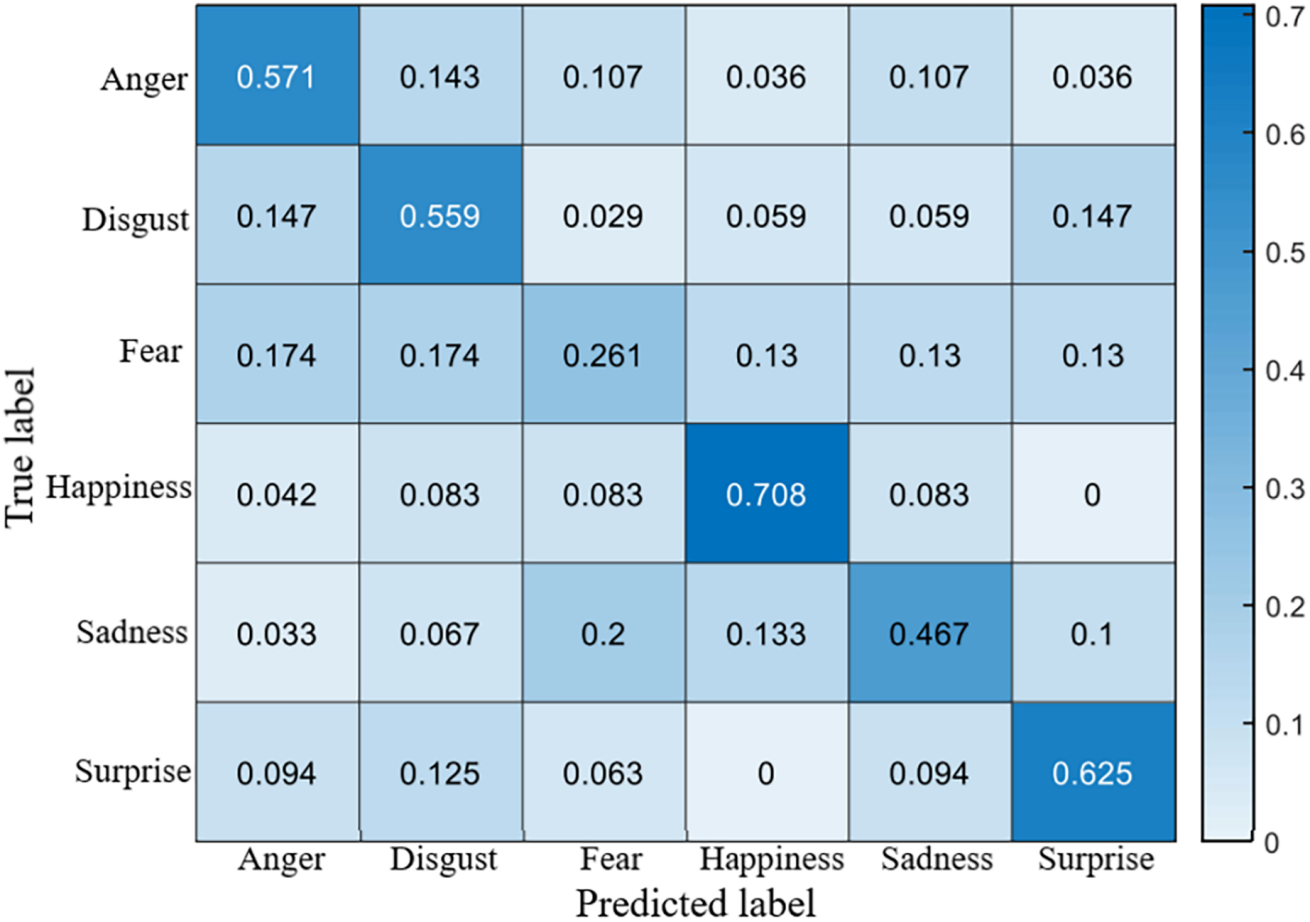
Confusion matrix on the SDU dataset.

**Figure 6:**
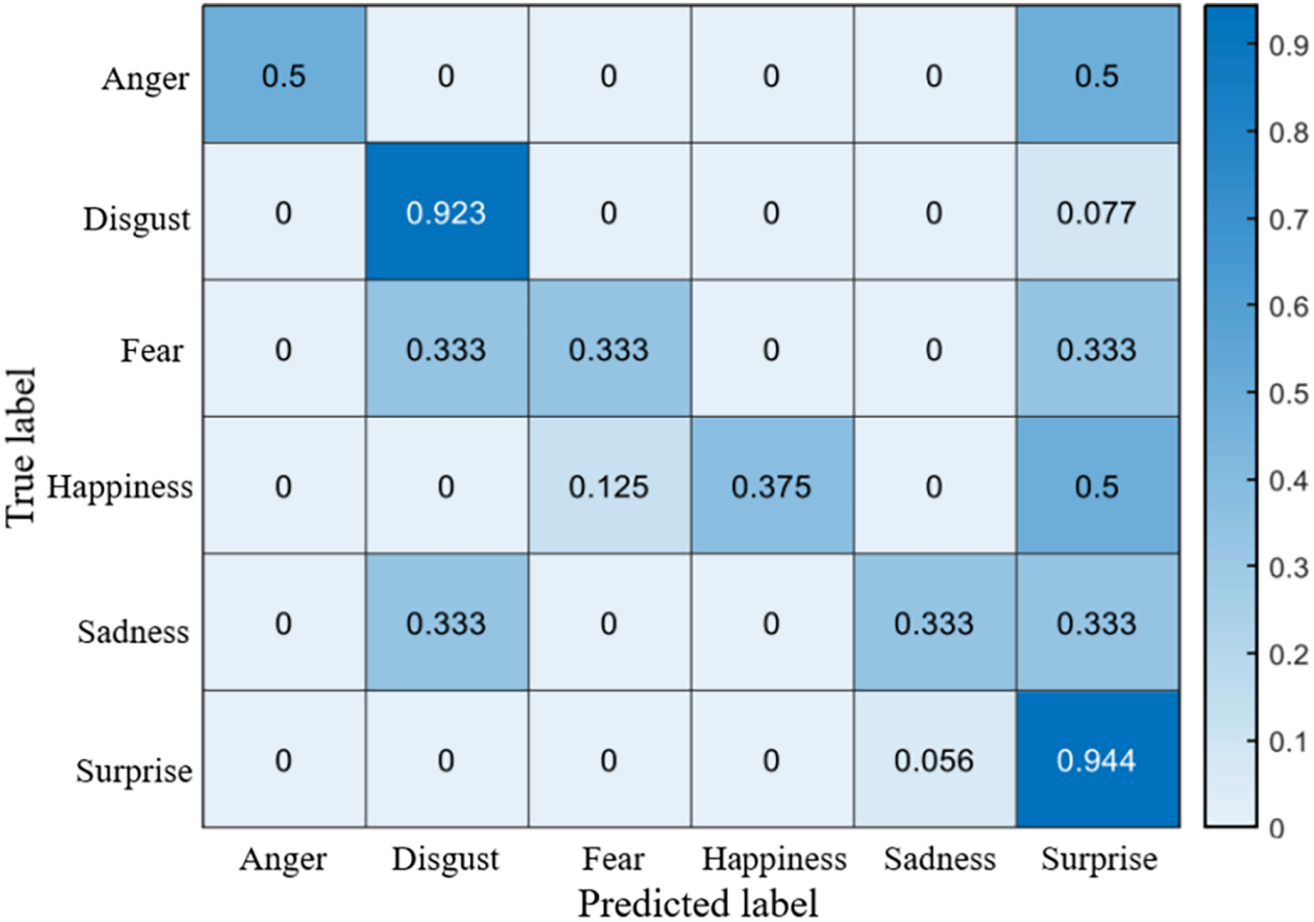
Confusion matrix on the MMEW dataset.

**Figure 7:**
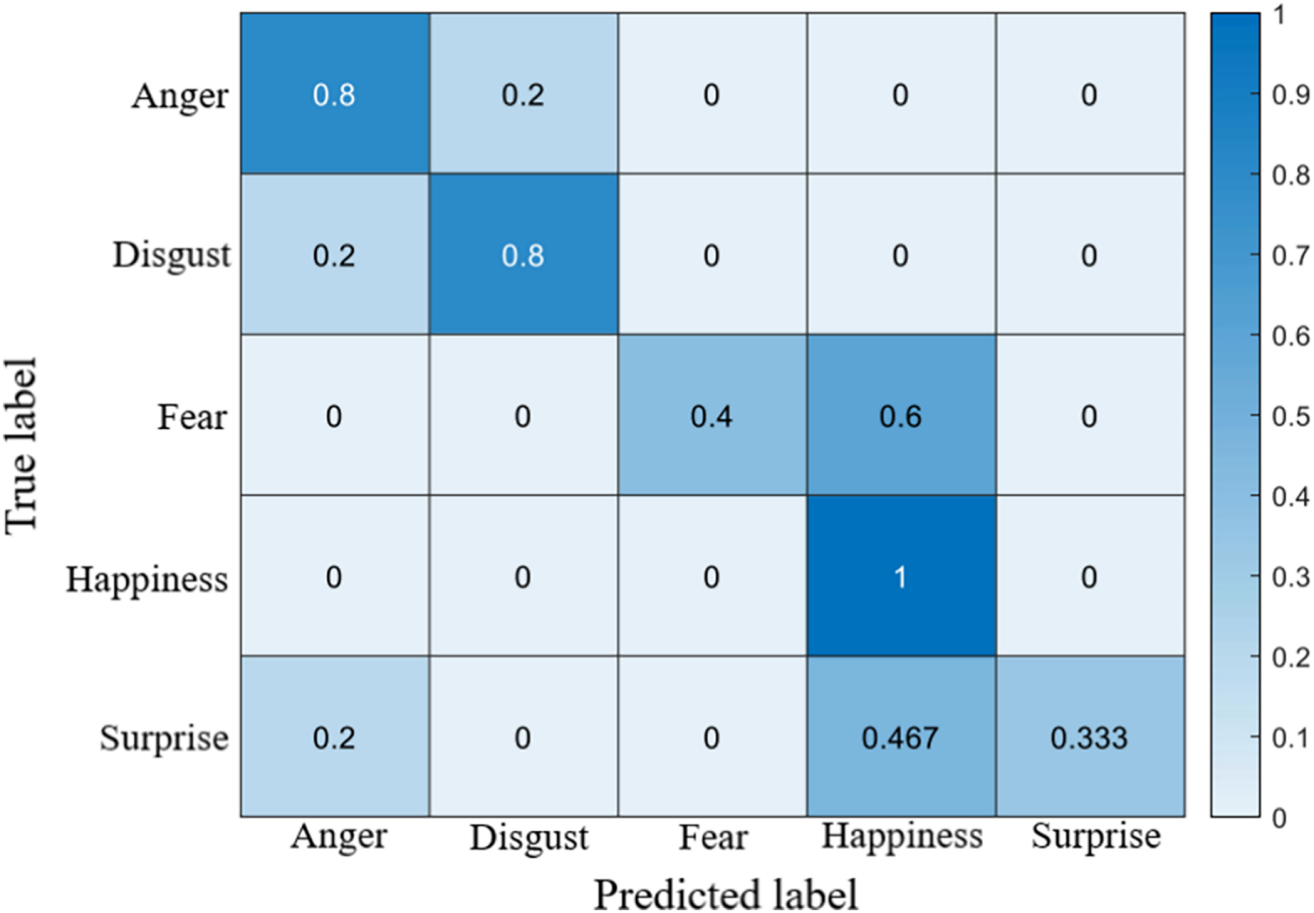
Confusion matrix on the SAMM dataset.

**Figure 8:**
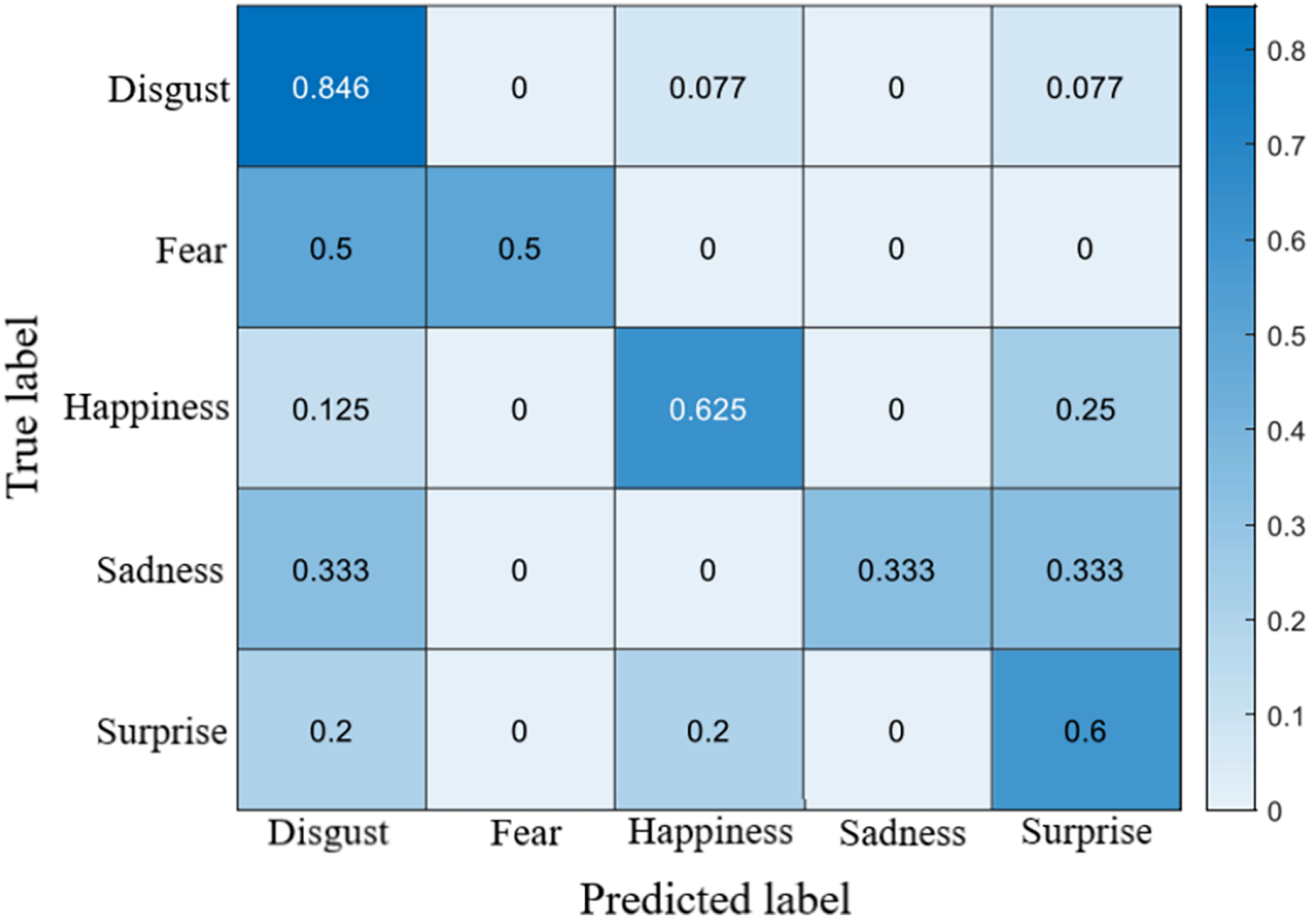
Confusion matrix on the CASME II dataset.

The recognition accuracy using our method on SDU, MMEW, SAMM and CASME II dataset is 53.9%, 70.3%, 71.4% and 70.7%, respectively. The confusion matrices of four experiments are demonstrated in Figs. 5–8, respectively.

In order to verify the effectiveness of DAO module, MINE module and different loss functions, comprehensive ablation experiments are conducted under different combinations of modules and loss functions on SDU dataset. The results are listed in Table 5.

**Table 5:** Ablation experiments.

No.	IDNet	MENet				Accuracy
	${L_{cross\_entropy\_d}}$	DAO	${L_{cross\_entropy\_e}}$	${L_{div}}$	${L_{MI}}$	
1			✓			39.9%
2		✓	✓			42.8%
3		✓	✓	✓		46.3%
4	✓		✓		✓	44.6%
5	✓	✓	✓		✓	48.7%
6	✓	✓	✓	✓	✓	**53.9%**

**Note:**

The highest result is marked in bold.

To better demonstrate the effectiveness of the proposed method, experiments using other existing methods are conducted to make a comparison. Five-fold cross-validation method is used for all experiments. The results are listed in Table 6.

**Table 6:** Comparisons with other methods.

Method	SDU	MMEW	SAMM	CASME II
FDM (Xu, Zhang & Wang, 2017)	19.7%	34.6%	34.1%	40.0%
LBP-TOP (Zhao & Pietikäinen, 2007)	33.8%	37.0%	38.9%	48.9%
MDMO (Liu et al., 2015)	44.7%	65.7%	50.0%	60.0%
Sparse MDMO (Liu, Li & Lai, 2021)	42.0%	60.0%	52.9%	64.4%
Transfer Learning (Peng et al., 2018)	37.6%	52.4%	55.9%	60.3%
ELRCN (Khor et al., 2018)	30.1%	41.5%	46.2%	55.6%
Graph-TCN (Lei et al., 2020)	51.0%	66.4%	61.4%	68.2%
RCN (Xia et al., 2020)	52.8%	62.6%	57.8%	64.7%
BDCNN (Chen et al., 2022)	51.4%	40.6%	41.7%	70.0%
Method in (Xie et al., 2020)	49.2%	63.2%	70.0%	60.5%
Ours	**53.9%**	**70.3%**	**71.4%**	**70.7%**

**Note:**

The highest results are marked in bold.

### Confusion matrices

It is clear that on SDU dataset the recognition accuracy of sadness, fear, anger and disgust is no more than 60%, while the accuracy of happiness reaches more than 70%. The reason is that sadness, fear, anger and disgust are all negative emotions, which leads to them being easily confused with each other. Fear has a lowest accuracy of only 26.1%, it is probably because that fear ME has too many facial movement regions, which may overlap the regions of other MEs.

On the MMEW dataset, disgust and surprise have a high accuracy of more than 90%, while the accuracy of other classes is relatively low. This is because in the MMEW dataset the disgust and surprise classes have a larger number of samples so that the model can better learn these two classes of ME.

On the SAMM dataset, like other datasets, the accuracy of fear is low. It is also worth noting that many surprise samples are predicted to be happiness, which is caused by the similar facial movement patterns of them.

On the CASMEII dataset, the accuracy of disgust is high, probably because of the distinctive motion pattern of it. In contrast, the accuracy of sadness and fear is relatively low due to a lack of samples.

### Results of ablation study

Experiments 1 and 2 show that the DAO module can improve the accuracy by 2.9%, which demonstrates that the DAO module can help the model obtain better performance. Experiment 2 and 3 show that by adding divergence loss to DAO module, the accuracy increases by 3.5%, which means that the divergence loss can help model obtain more discriminative features from different facial regions. From experiment 1 and 4 we can see that adding IDNet and MINE module improves the accuracy by 4.7%, which proves that decoupling identity features from ME features can effectively improve the recognition performance. However, it is worth noting that using MINE module alone performs worse than using only DAO module. Experiment 2, 4 and 5 illustrate that by using MINE module and DAO module together, a better result can be obtained, which again proves the effectiveness of both modules. Experiment 5 and 6 shows that adding divergence loss to DAO module improves the accuracy by 5.2%, which again proves the effectiveness of divergence loss function.

### Comparison with other methods

The recognition results of the proposed method are obviously better than other methods, which proves the superiority of the proposed algorithm.

## Discussion

The proposed method presents a novel idea for micro-expression recognition, namely decoupling the identity features from the learnt features to alleviate the negative impact of identity information on the recognition. In this study, the biggest interference of MER is analyzed and a novel deep model structure specially designed for this is proposed. Comprehensive experiments have demonstrated the effectiveness of the proposed method and have proved that decoupling identity features do help the deep model learn more discriminative micro-expression features to achieve a higher recognition rate. We hope this study can inspire future researches of MER. However, some problems still exist. Although the proposed IDNet can effectively learn identity information, the way of extracting identity features can still be explored. Moreover, the method of decoupling identity features from the ME features is not limited to the proposed one so further study can be conducted. Last but not least, although identity features are the biggest interferences, other redundant information still exists in ME video sequences such as light conditions and irrelevant facial parts and backgrounds. Further researches should be done to deal with these problems.

## Conclusions

To remove the redundant information in ME features extracted by deep model, a novel MER algorithm which decouples facial motion features and identity features is proposed in this paper. Based on the ResNet model, a dual branch neural network, which consists of MENet and IDNet, is designed to extract ME features and identity features. Global attention operation is used in IDNet to better extract the identity features. Multiple attention branches are used in MENet to help the model focus on different facial regions and extract more detailed ME features. A mutual information neural estimator is utilized to decouple facial motion features and identity features, which can help the model obtain more discriminative ME features. Experiments on SDU, MMEW, SAMM and CASME II datasets are conducted, which have achieved competitive results and proved the superiority of the proposed algorithm. Ablation study is also conducted, which has proved the effectiveness of each proposed module and each loss function. Although researchers have worked on it for decades, the MER tasks are still challenging. The current micro-expression datasets contain too much redundant information and interferences, which may be easily learnt by deep learning models. As a result, the key to promoting MER is to remove the redundant information from the extracted features. In future, new ways of extracting and decoupling identity features can be explored and the methods of alleviating other redundant information in micro-expression video sequences can be studied.

## Supplemental Information

10.7717/peerj-cs.1140/supp-1 Supplemental Information 1 Raw data of CASMEII dataset.

10.7717/peerj-cs.1140/supp-2 Supplemental Information 2 Raw data of SAMM dataset.

10.7717/peerj-cs.1140/supp-3 Supplemental Information 3 Raw data of MMEW dataset.

## Additional Information and Declarations

### Data Availability

The following information was supplied regarding data availability: The code is available at GitHub: https://github.com/TingxuanXie/DFMIDMER; TingxuanXie. (2022). TingxuanXie/DFMIDMER: v1.0 (v1.0). Zenodo. https://doi.org/10.5281/zenodo.7127169. The raw datasets are available in the Supplemental Files. We have used two third-party datasets: - SAMM dataset: http://www2.docm.mmu.ac.uk/STAFF/M.Yap/dataset.php. - CASME II dataset ©Xiaolan Fu: http://fu.psych.ac.cn/cn/Research/index.php.
